# Machine Learning-Driven Strength Prediction and Sustainability Analysis of Ultra-High-Performance Concrete

**DOI:** 10.3390/ma18225116

**Published:** 2025-11-11

**Authors:** Hongliang Rong, Wangwen Sun, Haoran Ma, Muhan Luo, Zhenghua You, Guobin Zhang, Pengcheng Zhu, Zhuangzhuang Liu, Lauren Y. Gómez-Zamorano

**Affiliations:** 1 Xinjiang Jiaotou Construction Management Co., Ltd., Urumchi 830000, China; 2 School of Highway, Chang’an University, Xi’an 710064, China; 3 Key Laboratory of Special Area Highway Engineering, Ministry of Education, Xi’an 710064, China; 4 International Joint Laboratory for Sustainable Development of Highway Infrastructures in Special Regions, Xi’an 710064, China; 5 Programa Doctoral en Ingeniería de Materiales, Facultad de Ingeniería Mecánica y Eléctrica, Universidad Autónoma de Nuevo León, Ave. Universidad s/n, Ciudad Universitaria, San Nicolás de los Garza 66455, Nuevo León, Mexico

**Keywords:** ultra-high-performance concrete, machine learning models, compressive strength prediction, sustainability assessment, environmental impact

## Abstract

Ultra-high-performance concrete (UHPC) is recognized for its exceptional strength and durability. However, the adoption of UHPC frequently leads to higher material and environmental costs. Accurate prediction of compressive strength is crucial for optimizing material design and reducing construction costs. In this study, a dataset of 800 samples was compiled from published articles. Four models, including random forest (RF), Gaussian Process Regression (GPR), Gradient Boosting (GB) and Artificial Neural Network (ANN), were applied. Results show that ANN and GPR achieved the best accuracy and stability. GB also performed well with good adaptability. RF captured general trends but produced larger errors in the high-strength range. Feature importance analysis highlighted curing age and cement content as the most influential factors, with a combined contribution above 65%. The water-to-binder ratio also affected strength through matrix densification. Extended evaluation with regression error characteristic (REC) curves and environmental impact index (EII) revealed the balance between performance and environmental impact. Higher compressive strength often required higher energy, CO_2_, and resource use. The range of 150–250 MPa showed a better balance between performance and sustainability. This study confirms the robustness of machine learning models for strength prediction and provides guidance for green and low-carbon ultra-high-performance concrete design.

## 1. Introduction

Concrete is the most widely used construction material worldwide, owing to its multiple engineering and economic advantages compared with alternative materials, including structural integrity, durability, modular construction, and cost-effectiveness [1,2]. It is composed of coarse aggregates, fine aggregates, water, and cementitious binders. To effectively evaluate the engineering performance of ultra-high-performance concrete under advanced design frameworks, its mechanical properties must be systematically investigated. Among these, compressive strength is the most critical factor [3]. As a core parameter in design and assessment, compressive strength is directly related to structural safety. It is relevant throughout the full life cycle of both new construction and existing structural evaluation. This property is influenced by multiple factors, such as particle size distribution, water-to-binder ratio, the proportion of recycled and natural aggregates, admixture dosage, and curing regime. Furthermore, it is closely linked to sustainability indicators. These indicators comprise material consumption, embodied carbon emissions, and energy demand [4,5].

Conventionally, physical experiments are the direct and authoritative approach to determining compressive strength. However, these experiments are often time- and resource-intensive. It is particularly inefficient to explore multiple mixtures and curing durations. Traditional regression-based methods have been employed to improve predictive efficiency. However, their capability is constrained by the inherent nonlinearity and high-dimensional interactions within the material structure system [2,6,7]. As a major branch of AI, ML excels at capturing complex relationships between input variables and outputs in large, heterogeneous, and noisy datasets. For ultra-high-performance concrete, predicting compressive strength has become a key regression task. Compared with conventional empirical regressions, ML methods can directly learn nonlinear mappings from multidimensional inputs such as material composition, processing conditions, and curing protocols. They often achieve higher predictive accuracy and stronger generalization [8,9].

A wide range of ML models have proven effective in capturing the nonlinear behavior of composite materials. On large tabular datasets, gradient boosting, random forests, and multi-model ensembles are frequently adopted as mainstream predictors of compressive strength [10]. The performance of these approaches, however, is heavily dependent on the representativeness, coverage, and quality of the training data. Recent research has extended the application of ML toward diverse ultra-high-performance concrete systems and alternative target properties, such as elastic modulus- and durability-related indicators [11,12].

Beyond mechanical performance, material selection and mixture design also directly affect environmental sustainability. The production of cementitious binders contributes substantially to greenhouse gas emissions and energy consumption. Recent studies have increasingly incorporated embodied CO_2_, energy consumption, and resource use into performance evaluation frameworks [13,14]. Accordingly, compressive strength prediction models based on experimental data or datasets are now progressively integrated with sustainability assessments. These approaches not only enhance predictive accuracy for mechanical properties but also identify trade-off regions between strength and environmental impacts. Therefore, it guides optimized mixture design. To further improve accuracy and robustness, hybrid ensemble models have been proposed. These models typically integrate multiple algorithms and representation learning layers to mitigate overfitting and bias drift [15,16,17].

This study is organized as follows. First, the research objectives and the machine learning (ML) approaches used are introduced. Then, the dataset is described. Statistical and correlation analyses are conducted to reveal variable interactions. The applied ML models are presented. Finally, predictive results are analyzed and discussed in conjunction with sustainability perspectives. Accurate prediction of compressive strength is not only essential for reliability but also represents a promising pathway to reducing experimental costs and minimizing carbon emissions [18,19]. Given the complex interactions among components in this heterogeneous material system, conventional empirical formulas and experimental methods are effective. However, they are inherently limited in generality and efficiency. The increasing use of green materials and low-carbon practices further accentuates these limitations. Fortunately, the latest advances in the field of machine learning offer new opportunities to improve predictive accuracy and integrate sustainability analysis. Gradient boosting algorithms have demonstrated remarkable performance in tabular prediction tasks and feature importance interpretation. Artificial neural networks perform well in capturing highly nonlinear interactions [20,21]. However, their standalone applications often struggle to balance accuracy with generalization. A hybrid framework combining these approaches can effectively exploit complementary strengths, thereby improving robustness and transferability.

In summary, methods including neural networks, random forests, and gradient boosting have been extensively explored. Some explainable tools further improve model transparency and reliability [22,23]. Recent advances in computer vision provide promising approaches to improve the data-driven modeling of ultra-high-performance concrete (UHPC). DeepLab enables the accurate semantic segmentation of microstructural features, while Efficient Net offers efficient and scalable feature extraction [24,25]. Integrating such vision-based frameworks could allow for the automatic identification of pores, fiber distributions, and crack networks, thereby improving the predictive accuracy and interpretability of UHPC performance models. Nevertheless, computational complexity and dependence on high-quality datasets remain significant challenges. Moreover, most existing studies focus primarily on mechanical performance, with insufficient consideration of embodied emissions, energy, and resource impacts. Although some investigations highlight the mechanical benefits of nanomaterials, their life-cycle environmental costs are rarely addressed. Similarly, while hybrid and automated ML approaches have achieved superior predictive accuracy, sustainability assessment remains underexplored [26,27]. To fill this research gap, the present work contributes an extended dataset that integrates traditional mixture proportions and curing ages with environmental features (CO_2_, energy, and resource consumption). Through multi-source data integration and stratified sampling strategies, this study boosts model robustness and generalizability. It provides an open benchmark for subsequent academic and industrial investigations.

## 2. Methodology and Research Approach

### 2.1. Data Preparation and Analysis

In this study, the dataset was systematically collected from experimental results reported in the existing literature. The final dataset comprises 800 sample records. Each record contains seven numerical parameters: cement content, superplasticizer, water, coarse aggregate, curing age, fine aggregate, and compressive strength. Among these, compressive strength is designated as the output variable to be predicted. The other six parameters are used as input features.

#### 2.1.1. Variable Description and Units

Table 1 provides detailed information on these variables, including their corresponding measurement units and brief descriptions of their roles in mix design. The analyses build a foundation for the subsequent analysis.

#### 2.1.2. Descriptive Analysis

According to the descriptive statistics presented in Table 2, the variables in the dataset exhibit varying degrees of variability and distributional characteristics. Cement content varies significantly across ultra-high-performance concrete mixtures. It has a mean of 348.56 kg/m^3^ and a standard deviation of 87.96 kg/m^3^. This reflects the diversity of mixture designs. The minimum and maximum values are 201.39 kg/m^3^ and 499.92 kg/m^3^, respectively, indicating a wide range of cement contents. Such variability may be associated with strength requirements, material types, and performance demands in ultra-high-performance concrete design. In contrast, water content demonstrates relatively high consistency, with a mean of 185.44 kg/m^3^ and a standard deviation of only 20.43 kg/m^3^. This suggests that water dosage is relatively stable. With limited variation, it ensures that workability can be maintained without significantly compromising strength. The dosage of superplasticizer varies more widely across mixtures, with a mean of 14.99 kg/m^3^ and a standard deviation of 8.71 kg/m^3^. This indicates that its usage differs depending on the requirements for workability and strength. Both coarse and fine aggregates exhibit relatively consistent usage levels. The mean values are 948.28 kg/m^3^ for coarse aggregates and 746.18 kg/m^3^ for fine aggregates. Their skewness values are close to zero. Their kurtosis values are negative, indicating symmetric and relatively flat distributions. Such distributions are favorable for the uniformity and stability of ultra-high-performance concrete mixtures, supporting the robustness of the overall structural integrity.

Curing age exhibits a high degree of variability, with a mean of 180.46 days and a standard deviation of 104.27 days. The range from 1 to 365 days indicates substantial differences in curing duration. It can significantly influence compressive strength. Longer curing time allow for more complete hydration of cement pastes, leading to greater strength. Finally, compressive strength has a mean value of 255.23 MPa. The skewness of 1.17 and kurtosis of 0.69 suggest that the distribution is slightly positively skewed and relatively flat. These characteristics may influence the accuracy of predictive modeling.

In summary, the descriptive statistical analysis highlights the variability of different variables in ultra-high-performance concrete mixtures. This provides valuable data for subsequent prediction and optimization. Statistics of the dataset lays a foundation for understanding the influence of mixture proportions and material properties on final performance [23,28].

#### 2.1.3. Data Visualization

**Figure 1:**
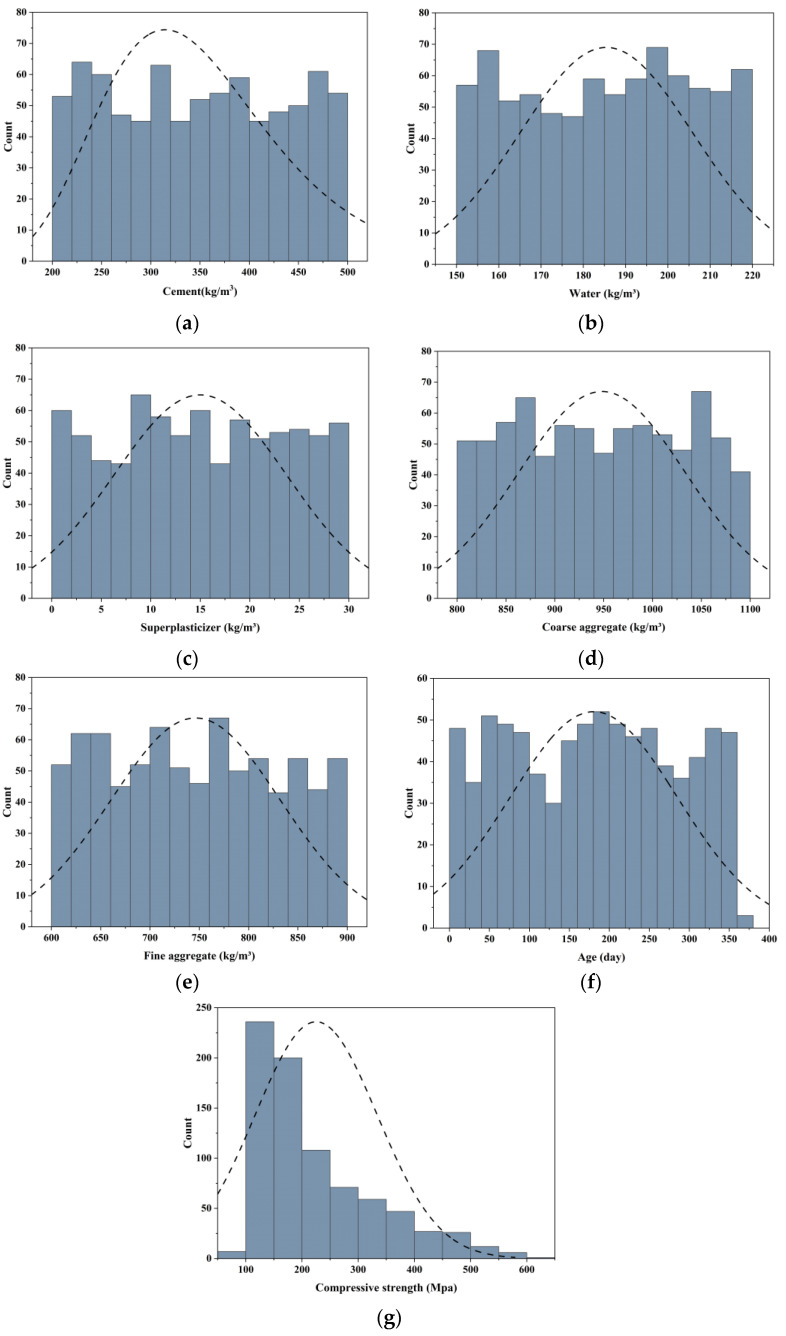
Histogram distributions of variables with normal curve fitting. (**a**) Cement; (**b**) Water; (**c**) Superplasticizer; (**d**) Coarse aggregate; (**e**) Fine aggregate; (**f**) Age; (**g**) Compressive strength.

Figure 1 illustrates the histogram distributions of the seven variables in the dataset, together with their fitted normal distribution curves. The variables cover comprehensive value ranges, ensuring the representativeness of the samples. Specifically, cement content is primarily distributed within 250–450 kg/m^3^, with a few samples exceeding 470 kg/m^3^. Although the overall histogram is relatively flat, the 320–380 kg/m^3^ range shows a clear concentration of samples, peaking at nearly 70. This indicates that experimental studies tend to focus on this common dosage range. Such a distribution highlights the sensitivity of compressive strength to cement dosage and provides strong training support for the model within medium dosage levels. Water content exhibits a relatively concentrated distribution, mainly between 160 and 210 kg/m^3^. The overall distribution closely follows a normal distribution, with the median being around 185–190 kg/m^3^, consistent with the water-to-binder ratio commonly found in ultra-high-performance concrete mixtures. Few samples fall below 160 kg/m^3^ or above 210 kg/m^3^, suggesting that extreme water-to-binder ratios are underrepresented. The distribution of superplasticizer dosage is more dispersed, spanning 0–25 kg/m^3^. Most samples fall within 5–15 kg/m^3^, with a peak of approximately 60–70 samples, whereas both lower and higher dosage regions contain fewer observations. This indicates that studies have placed greater emphasis on the effects of moderate dosages on workability and strength enhancement, while retaining some extreme cases that enable the model to capture nonlinear responses across dosage levels. Coarse aggregate content ranges from 850 to 1100 kg/m^3^. Fewer samples are found at both distribution tails, indicating limited investigation of extreme aggregate dosages. This distribution, relatively concentrated but with moderate spread, supports strong learning performance under typical aggregate contents while enabling limited extrapolation to boundary conditions. Fine aggregate content ranges between 650 and 950 kg/m^3^, with the majority concentrated in the 750–850 kg/m^3^ interval, peaking at nearly 70 samples. The number of samples decreases significantly above 900 kg/m^3^. Curing age shows a more typical distribution, ranging from 7 to 360 days, and approximates a normal shape. Substantial numbers of samples are present within both the 90–180 days range and at longer curing ages. This provides comprehensive information for predicting the compressive strength from early-age curing to long-term curing. Strength values are primarily concentrated between 100 and 200 MPa. The 100–150 MPa range accounts for the largest proportion, with more than 200 samples, forming the peak of the distribution.

In summary, the dataset exhibits reasonable coverage and distribution characteristics across all seven variables. High sample densities ensure reliable training accuracy under commonly adopted mixture conditions, while the inclusion of limited extreme cases broadens the data boundaries, thereby enhancing model robustness and generalization capacity.

**Figure 2:**
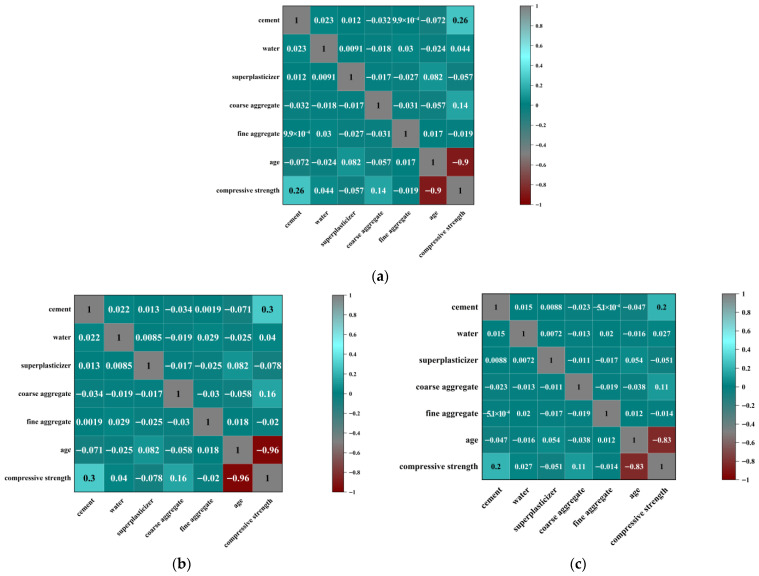
(**a**) Pearson’s correlation matrix; (**b**) Spearman correlation’s matrix; (**c**) Kendall correlation’s matrix.

In this study, Pearson, Spearman, and Kendall correlation coefficient matrices were plotted to provide a visual and quantitative analysis of the relationships between variables such as cement, coarse aggregate, fine aggregate, age, and the compressive strength of concrete. The three correlation measures are derived from different computational approaches, but their overall trends are consistent. This provides a robust quantitative foundation for subsequent analysis. In Figure 2a, the coefficient between cement and compressive strength is 0.26, indicating a weak but positive linear relationship. Coarse aggregate shows a coefficient of −0.032, nearly zero, suggesting almost no linear relationship with compressive strength. In contrast, curing age demonstrates a very strong negative linear correlation of −0.9, implying that as the curing period increases, the compressive strength significantly improves. Figure 2b presents similar but slightly stronger associations. Cement and compressive strength exhibit a coefficient of 0.3, suggesting a weak positive monotonic relationship, i.e., compressive strength generally increases with cement dosage. The coefficient between coarse aggregate and compressive strength remains low at −0.034, consistent with the Pearson result, confirming the absence of a meaningful monotonic association. Curing age, however, shows a much stronger negative monotonic correlation of −0.96, emphasizing the significance of strength gain with increasing curing time. This result also better captures potential nonlinear monotonic trends compared with Pearson. Figure 2c yields lower coefficients overall but maintains the same trends. Cement and compressive strength show a coefficient of 0.2, weaker than Spearman, suggesting a limited monotonic influence. Coarse aggregate remains nearly uncorrelated (−0.023). Meanwhile, curing age exhibits a strong negative monotonic correlation of −0.83, slightly weaker than Spearman but still confirming age as the dominant factor influencing compressive strength.

The analysis highlights curing age as the most influential factor in compressive strength development. Regardless of the method, the correlation coefficients consistently exceed |0.8|, confirming that compressive strength steadily increases with curing time and establishing age as a key determinant. By contrast, both fine and coarse aggregates exhibit negligible correlations, suggesting limited direct effects on compressive strength. The combined use of Pearson, Spearman, and Kendall coefficients serves a complementary role. Pearson is most suitable for detecting linear relationships, whereas Spearman and Kendall are better for capturing monotonic associations. The slightly higher Spearman coefficients relative to Kendall suggest the presence of nonlinear monotonic trends, while Kendall offers greater robustness to outliers. The consistent trends across all three methods enhance the reliability of the conclusions.

### 2.2. Development of ML Models

#### 2.2.1. Random Forest (RF) Method

Random Forest (RF) is a widely used ensemble learning method that develops a more robust and accurate predictive model by aggregating multiple independent decision trees [29,30]. RF introduces randomness in both sample and feature selection. At each split node, a subset of features is randomly selected, and the optimal split is identified. This dual-randomization strategy ensures diversity among decision trees, effectively mitigating overfitting and enhancing the robustness and adaptability of the predictive model.

**Figure 3:**
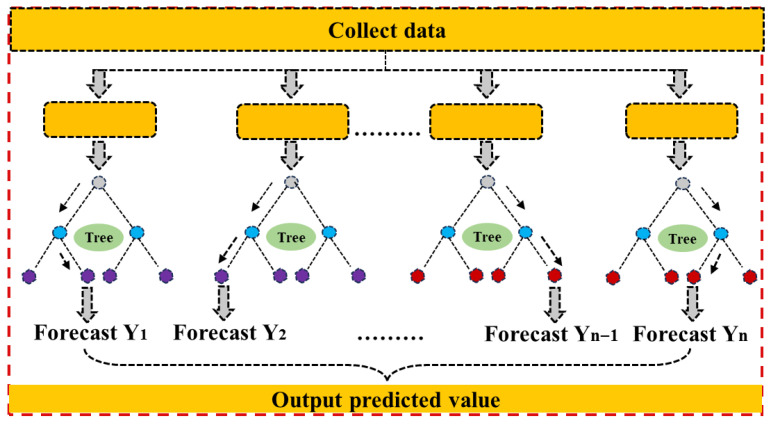
Workflow of random forest ensemble model.

As illustrated in Figure 3, the training process of RF can be divided into several steps. First, the original dataset is partitioned into multiple bootstrap samples, with each decision tree trained on a different subset. Second, at each splitting node, random feature selection is applied to determine the optimal threshold for data partitioning, and the tree is recursively expanded through left and right child nodes. An important parameter in this process is the number of trees included in the forest, which largely determines the stability and predictive accuracy of the model. In general, increasing the number of trees can effectively reduce variance and improve consistency in prediction results; however, an excessively large number of trees may also increase computational costs. Therefore, an appropriate balance must be achieved between predictive accuracy and computational efficiency.

#### 2.2.2. Gaussian Process Regression (GPR) Method

**Figure 4:**
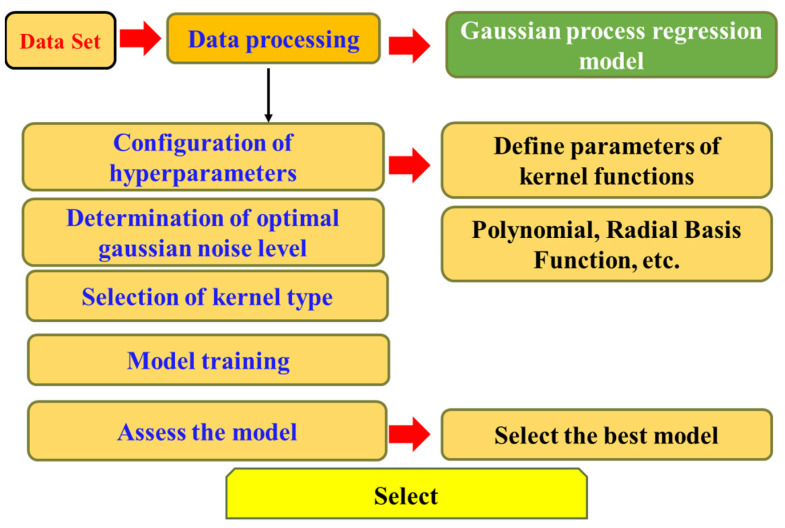
Workflow of gaussian process regression model.

Gaussian Process Regression (GPR) is a non-parametric supervised learning method widely used for its ability to capture complex nonlinear relationships [31,32]. The core idea is that adjacent observations often exhibit underlying correlations, which can be modeled by introducing a prior distribution in the function space. This formulation not only provides point predictions but also generates full predictive distributions corresponding to the test inputs, thereby enabling the regression analysis to capture both complex patterns and predictive uncertainty [33]. A crucial element in constructing the regression model is the choice of kernel function, which directly determines the model’s capacity to represent input relationships and its generalization ability. Various kernel functions have been proposed in the literature, including radial basis, exponential, and polynomial kernels. As illustrated in Figure 4, this study demonstrates the application of GPR to compressive strength prediction. By employing a probabilistic modeling approach, GPR not only provides estimates of the predicted values but also quantifies the uncertainty bounds associated with the predictions.

#### 2.2.3. Gradient Boosting (GB) Method

**Figure 5:**
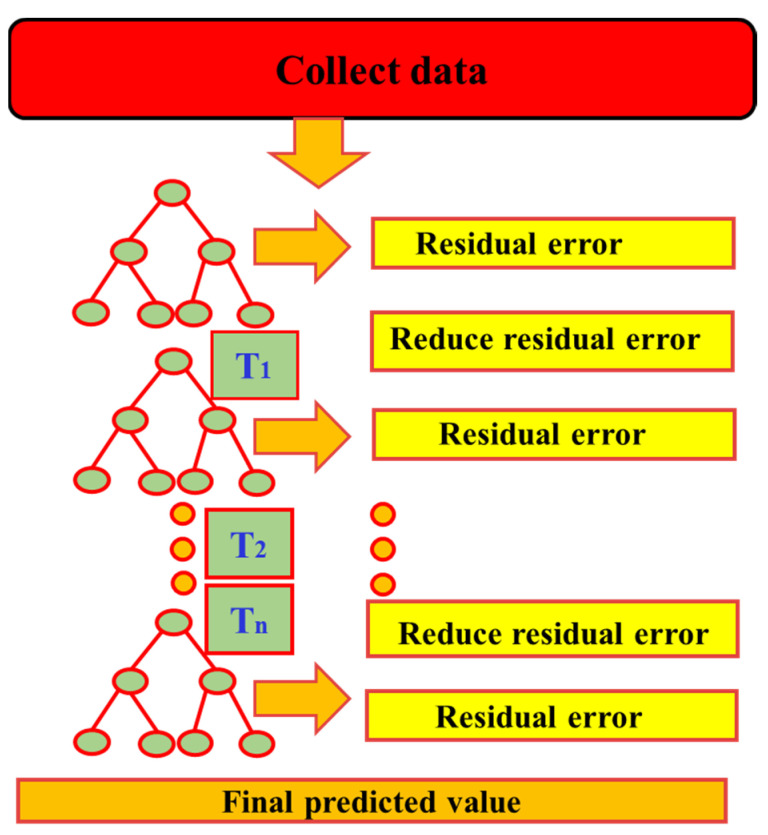
Workflow of gradient boosting model.

Gradient Boosting (GB) is an ensemble learning method based on gradient descent. Its fundamental idea is to iteratively improve the performance of weak learners so that, when combined, they form a powerful predictive model. At the initial stage, the algorithm begins with a base learner trained on the original dataset. Subsequently, residuals or errors are computed based on the predictions of the base model. By adjusting sample weights, misclassified or poorly predicted instances receive greater emphasis. After multiple iterations, all weak learners are aggregated through weighted combination to form a final model with strong predictive capability [34,35]. As illustrated in Figure 5, Gradient Boosting differs structurally from parallel tree-based ensemble methods such as Random Forests. While Random Forests enhance diversity by independently training multiple trees on different subsets of data and features, Gradient Boosting employs a sequential modeling strategy. Specifically, it leverages gradient information to iteratively optimize the loss function, with each step building upon the outcome of the preceding model. Due to its flexibility in both classification and regression tasks and its strength in capturing complex nonlinear relationships, Gradient Boosting has been widely recognized as an efficient and powerful ensemble learning method. It has found extensive applications across diverse machine learning and data modeling tasks.

#### 2.2.4. Artificial Neural Network (ANN) Method

**Figure 6:**
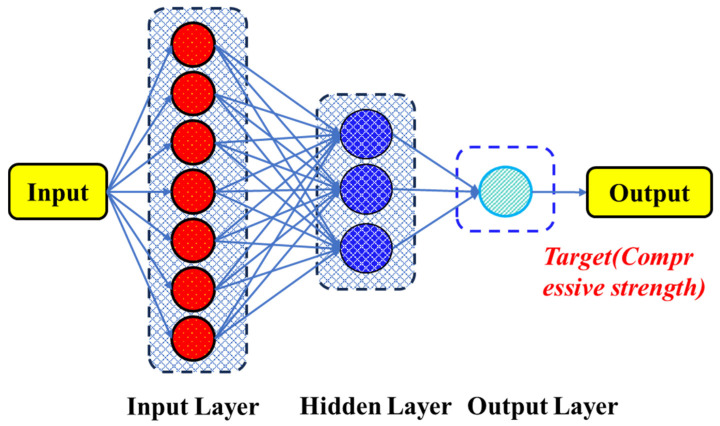
Workflow of artificial neural network model.

Artificial neural networks (ANNs) were conceived to simulate the mechanisms of perception and information processing in the human brain. As shown in Figure 6, each layer consists of numerous nodes (neurons) interconnected by weighted links, where the weights and bias terms jointly determine the strength and direction of information transfer across layers. In operation, the input layer receives external data and transmits it to the hidden layers. Within the hidden layers, neurons compute the weighted sum of the incoming signals, add a bias term, and then apply a nonlinear activation function to the result. This process allows the network to extract latent patterns and features, thereby establishing complex nonlinear mappings between the input and output spaces [35,36]. Finally, the output layer aggregates the results of the previous computations to generate predictions. Specifically, the ANN model consisted of one input layer, three hidden layers, and one output layer. The input layer received all normalized mixture parameters. The three hidden layers contained 64, 32, and 16 neurons, respectively. Each hidden layer adopted the Rectified Linear Unit activation function to introduce nonlinearity, accelerate convergence, and prevent vanishing-gradient problems, while the output layer employed a linear activation function suitable for continuous variable prediction. The model was trained using the Adam optimizer with an appropriate learning rate and batch size. Early stopping and dropout regularization were applied to reduce overfitting and to maintain the network’s generalization capability. Model training and validation were conducted through a 10-fold cross-validation process, ensuring statistical robustness and performance stability across different data partitions. During training, the ANN employs the backpropagation algorithm to iteratively update weights and biases in order to minimize the loss function, thereby progressively improving model performance. Once the predefined convergence criteria are met, the training process terminates, and the model can be used for practical prediction tasks. By incorporating nonlinear activation functions, ANN possess strong representational power, enabling them to learn and capture complex nonlinear relationships. This makes them highly adaptable and flexible across various domains, including regression analysis, classification, and pattern recognition. Moreover, ANN exhibit good generalization capability, providing relatively accurate predictions on previously unseen data after being trained on representative datasets.

#### 2.2.5. Prediction Framework

**Figure 7:**
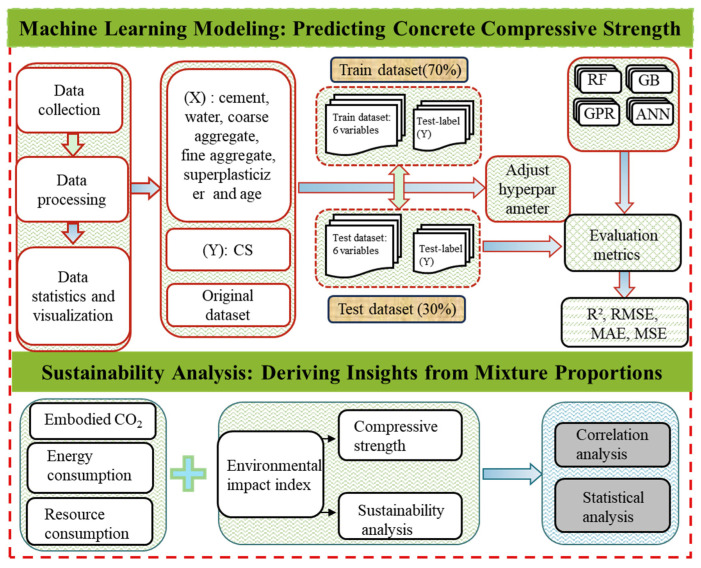
Framework for ultra-high-performance concrete performance prediction and sustainability assessment.

The overall research framework of this study is illustrated in Figure 7. It consists of two main components: machine learning modeling of ultra-high-performance concrete compressive strength and sustainability analysis. Data collection and preprocessing are conducted. The process includes data cleaning and normalization as well as statistical and visualization analyses. These steps ensure the representativeness and reliability of the dataset. The input variables encompass typical mixture parameters of ultra-high-performance concrete, including cement, water, coarse aggregate, fine aggregate, admixture, and curing age. While the output variable is compressive strength (CS). Several machine learning models, including Random Forest (RF), Gradient Boosting (GB), Gaussian Process Regression (GPR), and Artificial neural network (ANN), are trained and evaluated. During model training, hyperparameter tuning is applied to optimize model performance. Prediction results are systematically assessed using metrics such as R^2^, RMSE, MAE, and MSE to ensure accuracy and robustness. This study integrates mechanical performance with environmental impact for sustainability analysis. Specifically, embodied CO_2_ emissions, energy consumption, and resource use associated with ultra-high-performance concrete production and application are considered to construct a comprehensive environmental impact index. By combining this index with the predicted compressive strength, ultra-high-performance concrete performance evaluation is extended beyond mechanical properties to also include environmental and resource dimensions. Furthermore, correlation and statistical analyses are performed to systematically examine the combined effects of mixture proportions on compressive performance and environmental impact. Overall, the framework builds a clear connection between data-driven prediction and environmental evaluation. This integration provides scientific evidence and decision support for the design and optimization of green and low-carbon ultra-high-performance concrete.

### 2.3. Evaluation Metrics for Prediction Performance

In this study, model performance was evaluated using multiple statistical metrics, including Mean Squared Error (MSE), Root Mean Squared Error (RMSE), Mean Absolute Error (MAE), and the coefficient of determination (R^2^) [37,38]. Their mathematical formulations are provided in Equations (1)–(4). Each metric emphasizes different aspects of predictive performance. MSE measures the average squared difference between predicted and actual values, reflecting the overall level of deviation. As it penalizes larger errors more heavily, MSE is particularly sensitive to poor predictions on extreme samples. As the square root of MSE, RMSE provides a more interpretable measure of error magnitude while maintaining high sensitivity to large deviation. In contrast, MAE quantifies the mean absolute difference between predicted and observed values, providing an easily interpretable measure of prediction error. Finally, R^2^ evaluates the proportion of variance in the observed data that is explained by the model, where values approaching 1 denote a better fit. By combining these complementary evaluation metrics, the model’s goodness of fit and robustness can be analyzed from multiple perspectives, ensuring that the assessment results are both comprehensive and reliable.

- Mean Squared Error:

(1) $$RMSE=\sqrt{\frac{\sum_{i=1}^{n}(Z_{i}-{\overset{\wedge }{Z}}_{i}{)}^{2}}{n}}$$

- Mean Absolute Error:

(2) $$MAE=\frac{\sum_{i=1}^{n}\left|Z_{i}-{\overset{\wedge }{Z}}_{i}\right|}{n}$$

- Coefficient of determination:

(3) $$R^{2}=1-\frac{\sum_{i=1}^{n}{\left({\overset{\wedge }{Z}}_{i}-Z_{i}\right)}^{2}}{\sum_{i=1}^{n}{\left(Z_{i}-\bar{Z}\right)}^{2}}$$

- Root Mean Squared Error:

(4) $$MSE=\frac{\sum_{i=1}^{n}(Z_{i}-{\overset{\wedge }{Z}}_{i}{)}^{2}}{n}$$

Note: In these formulations, $Z_{i}$ and ${\overset{\wedge }{Z}}_{i}$ denote the *i*-th measured and model-predicted values, respectively, and *n* corresponds to the number of values included in the dataset.

## 3. Results and Discussion

### 3.1. Statistical Assessment of Compressive Strength Prediction Models

**Figure 8:**
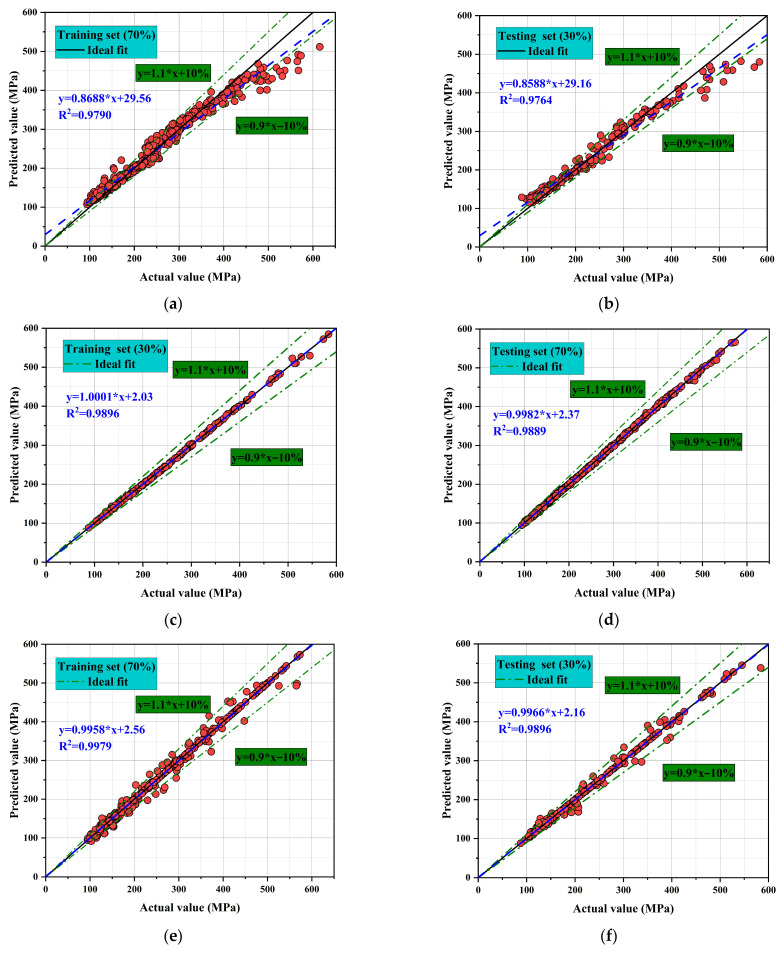

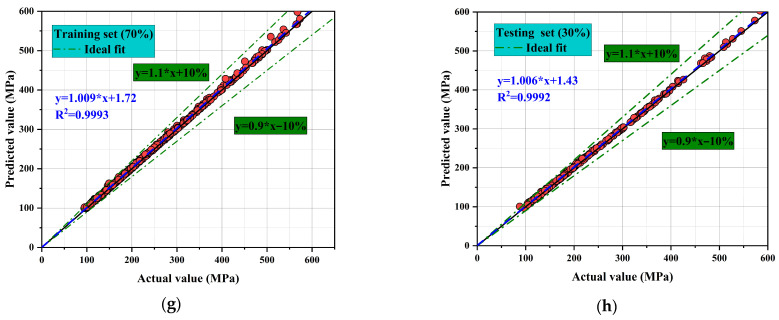
Comparison of compressive strength predictions from different machine learning models. (**a**) RF (Training set); (**b**) RF (Testing set); (**c**) GPR (Training set); (**d**) GPR (Testing set); (**e**) GB (Training set); (**f**) GB (Testing set); (**g**) ANN (Training set); (**h**) ANN (Testing set).

Figure 8 presents the fitting performance of four machine learning models, including Random Forest (RF), Gaussian Process Regression (GPR), Gradient Boosting (GB), and Artificial neural network (ANN), on both training and testing datasets. Overall the predictions align closely with the observations. Most points lie near the ideal line (y = x) and within ± 10% error, indicating reliable compressive-strength prediction across all models. However, differences in prediction accuracy and stability are evident across models. GPR and ANN demonstrate the closest fits, with R^2^ values approaching 1 on the testing set, reflecting superior generalization capability. I In contrast, RF and GB show good overall trends but exhibit larger deviations in some cases, indicating limited capacity to handle extreme values [39,40].

For the RF model, the coefficient of determination reaches R^2^ = 0.9790 for the training set and R^2^ = 0.9764 for the testing set. The corresponding regression equations are y = 0.8688x + 29.56 and y = 0.8588x + 29.16, respectively. The slopes being less than 1 indicate an overall underestimation trend, particularly pronounced in the high-strength region. This suggests that while RF captures the general trend. It lacks sufficient accuracy under extreme conditions [41]. The GPR model exhibits outstanding precision. For the training set, R^2^ = 0.9896 with a regression equation of y = 1.001x + 2.03. For the testing set, R^2^ reaches as high as 0.9889 with y = 0.9982x + 2.37, nearly perfectly coinciding with the ideal line. All points fall within the ± 10% interval, confirming excellent generalization performance and minimal signs of overfitting or underfitting. The GB model performs slightly less favorably. The training set yields R^2^ = 0.9958, while the testing set yields R^2^ = 0.9966. The regression equations have slopes close to 1, and most points lie within the ±10% error interval. Although overall accuracy is comparable to GPR, minor deviations are observed in the high-strength region, indicating weaker performance in handling extreme data. The ANN model demonstrates the best performance among all. Predicted and actual values almost perfectly overlap, with all points tightly distributed along the ideal line and within the ±10% interval. This confirms that ANN possesses a unique advantage in capturing complex nonlinear relationships, enabling highly accurate predictions of compressive strength.

The overall ranking of model performance is: ANN = GPR > GB > RF. ANN achieves optimal fitting through its strong nonlinear mapping capability. While GPR stands out for its probabilistic framework that ensures both predictive accuracy and uncertainty quantification. GB shows high accuracy but limited performance in high-strength regions. RF reflects overall trends but is less precise for extreme data. Mechanistically, ANN applies multiple layers and nonlinear activation functions to capture complex input–output relationships. These characteristics account for its superior predictive performance. GPR excels by combining predictions with uncertainty estimation, making it well-suited for high-confidence forecasting tasks. GB relies on iterative boosting of weak learners, yielding reasonably high accuracy but greater sensitivity to noise. RF, with its random feature selection, reduces overfitting risk but struggles with complex nonlinear problems [42,43]. Consequently, ANN and GPR are identified as the most suitable models for predicting compressive strength.

**Figure 9:**
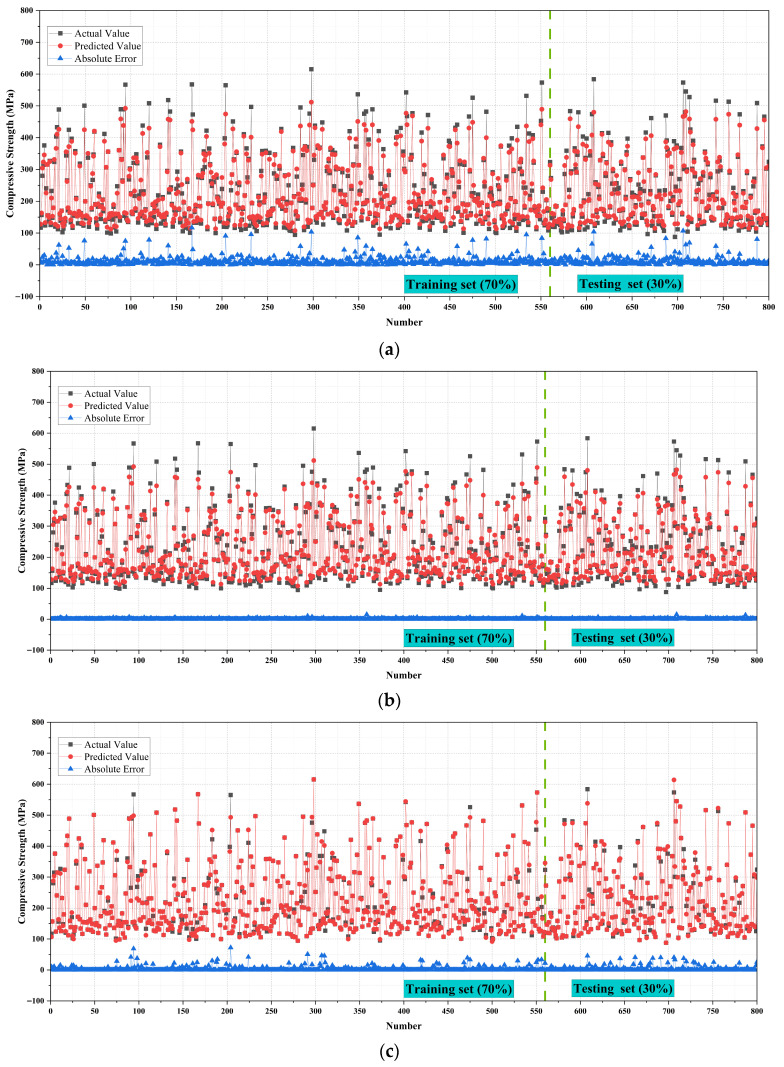

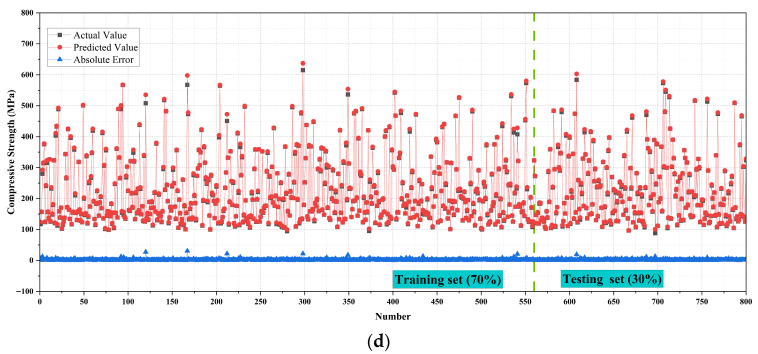
Fitting performance and error distribution of different models for compressive strength prediction. (**a**) RF; (**b**) GPR; (**c**) GB; (**d**) ANN.

Figure 9 illustrates the actual values, predicted values, and absolute error distributions for the four machine learning models in predicting compressive strength. Black dots represent actual values, red dots denote predicted values, and blue lines indicate the absolute prediction errors. The dataset was divided into 70% for training and 30% for testing, separated by a green dashed line. Overall, all four models fit the actual strength distribution reasonably well, yet notable differences remain in terms of prediction accuracy and error control.

For the RF model, the predicted values generally follow the actual trend but display substantial dispersion. In several cases, prediction deviations exceed ±50 MPa, with error fluctuations particularly pronounced in the high-strength region. This indicates that while RF can capture overall trends in compressive strength, it struggles with complex nonlinear relationships, thereby limiting its generalization ability. The GPR model demonstrates more stable and accurate performance. Most predicted values closely overlap the actual values. The majority of errors are within ±20 MPa, with only a few points exceeding this range. The overall error curve lies consistently below that of RF, suggesting that GPR can more effectively model the nonlinear relationships between compressive strength and input variables. It also maintains strong consistency and generalization between training and testing sets [44,45].

The GB model shows intermediate predictive performance. Its overall fitting results are good, with most prediction errors controlled within ±30 MPa. However, some samples still exhibit larger deviations. Compared with GPR, the error distribution of GB is more dispersed, indicating that although the model performs reliably across most ranges, its accuracy decreases when handling extreme values. The ANN model delivers the best predictive performance. Predicted values almost perfectly coincide with actual values, with red and black dots highly overlapping. The blue absolute error curve is nearly aligned with the zero line, with minimal fluctuation. For the vast majority of samples, The absolute error remains below ±10 MPa, markedly lower than those of RF, GB, and GPR. This demonstrates that ANN can fully capture the complex nonlinear interactions among input features and exhibits both high consistency and excellent generalization across training and testing sets [46,47].

In summary, ANN stands out for its extremely low absolute errors and superior nonlinear learning capacity. GPR also shows strong precision and robustness, with consistently small errors. RF captures the overall trend but suffers from insufficient predictive accuracy and stability.

### 3.2. Application of REC Curves in Evaluating Compressive Strength Prediction Models

**Figure 10:**
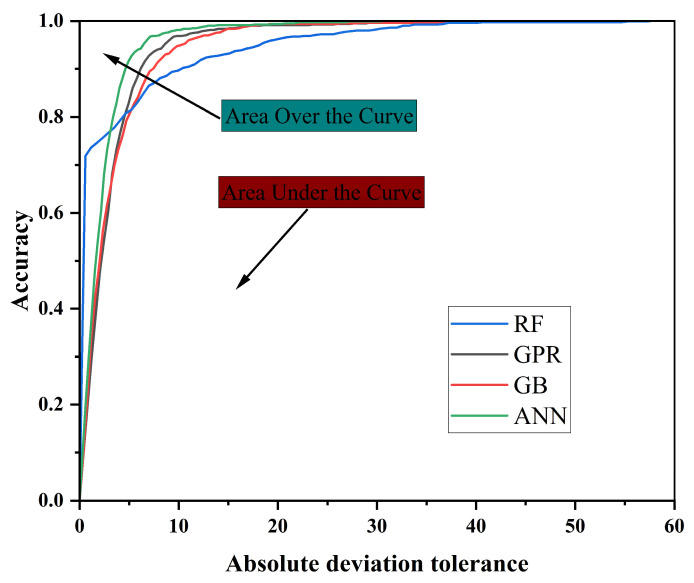
Analysis of regression error characteristic (REC) curves.

The Regression Error Characteristic (REC) curve has emerged in recent years as an important tool for evaluating and comparing the predictive ability of regression models. It provides an intuitive visualization of how the accuracy of predictions varies under different absolute error tolerance levels. Figure 10 presents the REC curves of the four machine learning models. It clearly reveals their performance differences across varying tolerance intervals and offering a straightforward basis for assessing predictive accuracy and stability [48].

As shown in Figure 10, the curves of GB and ANN rise the fastest. When the error tolerance is below 10, their accuracy already exceeds 90%, and both curves approach 100% rapidly at around a tolerance of 20. This indicates that these two models can achieve high predictive accuracy within very small error bounds, reflecting strong generalization capability and fast convergence. The GPR model ranks slightly lower; although its curve rises more slowly than those of GB and ANN, it still achieves relatively high accuracy under low error tolerance and maintains stability in the middle and later stages, highlighting its robustness and adaptability to uncertainty [49,50]. By contrast, the RF model curve increases much more slowly. Within low error tolerance ranges, its accuracy consistently lags behind the other three models, only approaching 100% when tolerance expands beyond 30. This demonstrates that RF has limited predictive ability within small error ranges.

The Area Over the Curve (AOC) is a key metric for assessing overall REC performance, as it reflects the cumulative distribution of prediction errors [51]. Ideally, a perfect regression model should have a very small AOC value, with its curve running nearly parallel to the *y*-axis, reaching high accuracy at minimal error tolerance. Based on Figure 10, GB and ANN achieve the lowest AOC values, indicating the best overall performance. GPR follows closely, and although it is slightly less competitive at extremely low error tolerances, its stability and narrow error distribution still reflect high reliability. In contrast, RF shows the highest AOC value, meaning its cumulative error is the largest and its overall predictive accuracy is the weakest.

Further analysis clarifies the performance differences among models. The GPR curve is the steepest, showing that it can achieve high prediction accuracy even at very low error tolerance, thereby underscoring its reliability and robustness [52]. By comparison, GB and ANN demonstrate higher overall accuracy but their curves are relatively flatter. Their advantage is less pronounced in some tolerance ranges. The RF curve is the flattest, indicating greater prediction uncertainty and lower accuracy and stability compared to the other models.

**Figure 11:**
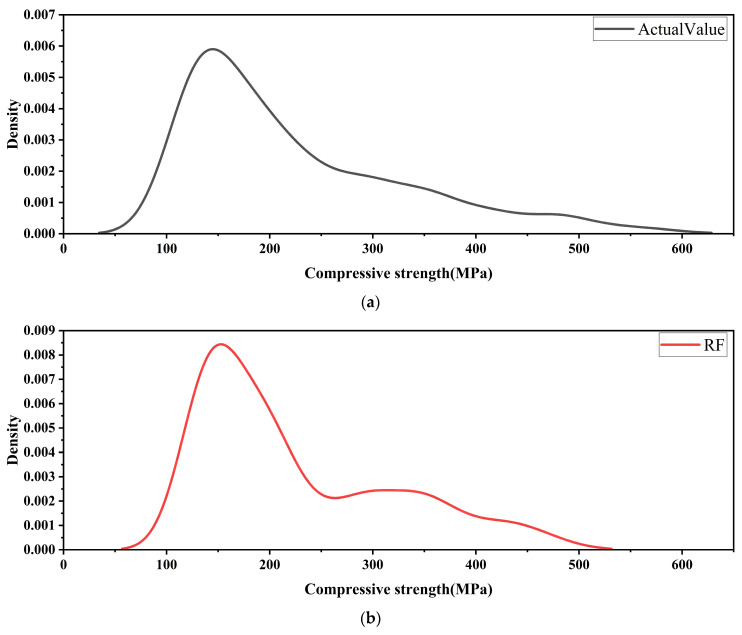

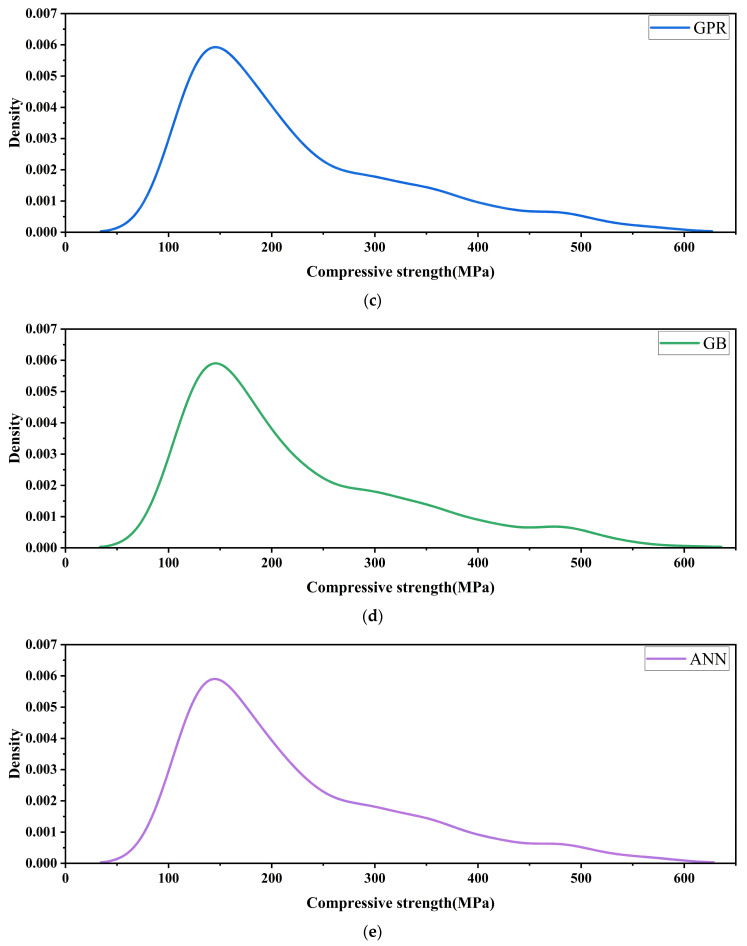
Statistical analysis of density distribution for predicted and actual compressive strength. (**a**) Actual Value (**b**) RF; (**c**) GPR; (**d**) GB; (**e**) ANN.

Figure 11 compares the distribution of actual compressive strength with the prediction error distributions of different machine learning models, providing an intuitive reflection of their stability in predicting ultra-high-performance concrete compressive strength.

As shown in Figure 11a, the actual compressive strength exhibits a unimodal distribution. The highest probability density peak is in the 150–250 MPa range, approximating a normal distribution. This interval represents the typical strength level of most specimens, making accurate prediction in this range particularly important. In Figure 11b, the error distribution of the Random Forest (RF) model is highly dispersed. Although the main peak is located close to that of the actual strength distribution, its height is lower, and the tail is extended, resulting in a broad error interval [53]. This indicates that RF can capture the general trend but tends to produce large deviations under high-precision requirements, limiting its stability and confidence in predictions. Figure 11c illustrates the results of the Gaussian Process Regression (GPR) model. Its error distribution aligns almost perfectly with the actual strength distribution: the main peak nearly overlaps with the actual data, the peak height is comparable to the maximum density of the actual distribution, and the tails converge smoothly. This demonstrates that GPR maintains stable predictions within narrow error ranges, with strong generalization capability and low uncertainty, highlighting its superiority in regression tasks. In Figure 11d, the Gradient Boosting (GB) model also shows strong fitting capability. Its main peak closely matches the actual distribution with a relatively high intensity, though the tails are somewhat wider than those of GPR. This suggests that GB delivers excellent overall predictive accuracy but is slightly less adaptive to extreme samples, leading to occasional uncertainty fluctuations [54]. Figure 11e shows the Artificial Neural Network (ANN) model results, which fall between GPR and GB. The error distribution trend is consistent with the actual strength distribution, and the main peak lies within the correct range, adequately capturing the core characteristics of compressive strength. However, the peak height is slightly lower than that of GPR and GB, while the distribution range is broader, with more pronounced tail stretching. This indicates that although ANN can capture the main distribution, its predictions exhibit certain fluctuations across samples, making its stability inferior to that of GPR [55,56].

Overall, the GPR model shows the most concentrated error distribution and its predictions closely match the experimental values. This indicates superior accuracy and stability. GB ranks second, performing strongly overall but with limited error control for individual samples. ANN demonstrates good predictive capability, successfully fitting the main distribution but with weaker error convergence, implying greater variability in results. RF performs the weakest, with widely dispersed errors, making it less suitable for high-precision prediction tasks. While ANN does not match the precision and robustness of GPR, it still demonstrates strong predictive ability and remains a reliable modeling approach, particularly suitable for scenarios requiring strong nonlinear fitting capacity [57].

### 3.3. K-Fold Cross Validation Analysis

To ensure a balance between training effectiveness and predictive performance, the dataset was first divided into training and testing sets at a ratio of 70% to 30%. A 10-fold cross-validation strategy was further employed to assess model robustness. The training set (70%) was used for model learning and parameter optimization. The testing set (30%) was used as an independent dataset to assess the generalization capability of the trained models.

**Figure 12:**
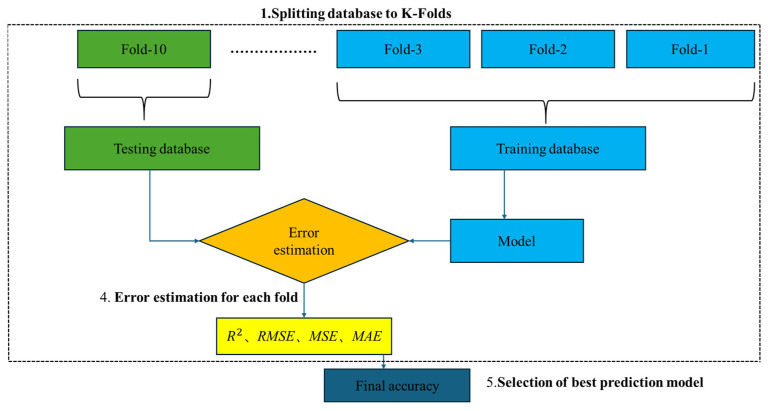
Training–testing data partitioning and model validation procedure.

As illustrated in Figure 12, the training set was equally partitioned into 10 approximately equal subsets (Fold-1 to Fold-10). In each iteration, one subset served as the validation set, and the remaining nine were used to train the model [58]. After training, the model was applied to the validation set to generate predictions and compute the corresponding error metrics. This process was repeated 10 times, ensuring that each subset was used exactly once as the validation set. Such an approach maximizes the utilization of the training data and minimizes the risk of random bias caused by a single partition. For performance evaluation, multiple statistical metrics were employed [59,60], including the coefficient of determination (R^2^), root mean squared error (RMSE), mean squared error (MSE), and mean absolute error (MAE), providing an intuitive quantification of prediction error. The reported performance metrics represent the average values across ten folds, and the variability among folds was minimal, confirming the overall stability of the models. For brevity, only mean results are shown.

**Figure 13:**
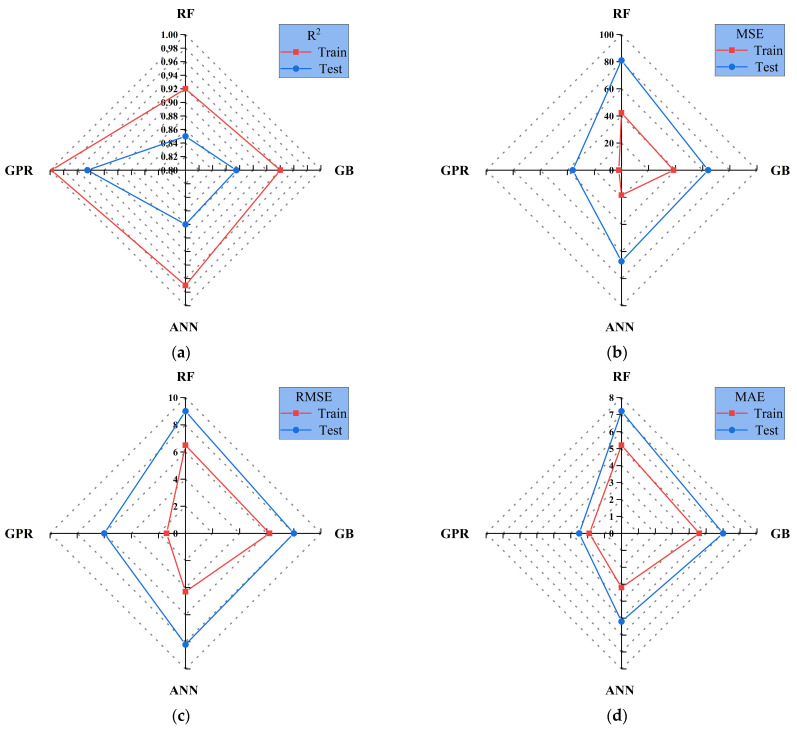
Radar chart of different models across multiple evaluation metrics. (**a**) R^2^; (**b**) MSE; (**c**) RMSE; (**d**) MAE.

Figure 13 compares the evaluation metrics of the four machine learning models on both training and testing sets using radar plots, providing a more intuitive comparison of their fitting ability and generalization performance.

As shown in Figure 13a, the R^2^ values for RF, GB, and ANN in the training set all exceed 0.92, indicating strong fitting ability. Specifically, RF achieves an R^2^ of approximately 0.95, ANN reaches close to 0.96, while GB is slightly lower at about 0.93. By contrast, GPR records a lower R^2^ of around 0.90. In the testing set, RF drops to 0.82, showing instability. ANN decreases to about 0.84, indicating overfitting. GPR and GB keep higher R^2^ values of 0.88 and 0.90, showing better generalization [61,62]. Figure 13b presents the MSE results. In the training set, ANN and GB achieve the lowest MSE values (approximately 15 and 18, respectively), demonstrating high fitting accuracy. RF is slightly higher at around 25, while GPR records an MSE of about 20. In the testing set, GPR and GB maintain relatively low MSE values (30–35), indicating controlled errors on independent data. By contrast, ANN rises sharply to about 50. RF performs the worst, reaching nearly 80 and showing clear error accumulation. Figure 13c shows the RMSE values. In the training set, ANN records the lowest RMSE of about 3.9, followed by GB at 4.2, GPR at 4.5, and RF at 5.0. In the testing set, GPR remains the most stable with an RMSE of around 5.5, while GB rises slightly to about 6.0. ANN increases significantly to 7.2, and RF reaches the highest level, approaching 9.0–10.0, indicating substantial prediction variability [37]. Figure 13d reports the MAE results. In the training set, ANN achieves the lowest MAE of around 2.5, while GB and GPR record values of 2.8 and 3.0, respectively. RF is slightly higher at approximately 3.5. In the testing set, GPR and GB maintain relatively low MAE values of 3.8 and 4.0, respectively. In contrast, ANN increases to 5.0, while RF records the highest MAE of nearly 7.0–7.5. Overall, the four machine learning models exhibit distinct performance differences in predicting concrete compressive strength. The GPR model performs best. It reaches an R^2^ of 0.88 on the testing set and keeps low RMSE and MAE, showing good generalization. GB performs well and ranks second. It keeps R^2^ around 0.90 and moderate errors, showing good transferability [63,64]. The ANN model performs best on the training set (R^2^ = 0.96, RMSE = 3.9, MAE = 2.5). But its performance declines significantly on the testing set (R^2^ = 0.84, RMSE = 7.2, MAE = 5.0), indicating overfitting. The ANN model showed slight overfitting as the training R^2^ was 0.96 and the testing R^2^ was 0.84. This result was mainly caused by the small dataset size and the high capacity of the neural network. Early stopping and dropout regularization were used to reduce overfitting. RF performs reasonably well in the training set (R^2^ = 0.95). However, the R^2^ on the testing set drops sharply to 0.82. The error metrics are the highest (MSE ≈ 80, RMSE ≈ 10, MAE ≈ 7.5), showing the weakest performance.

### 3.4. Quantitative Analysis of Feature Importance

**Figure 14:**
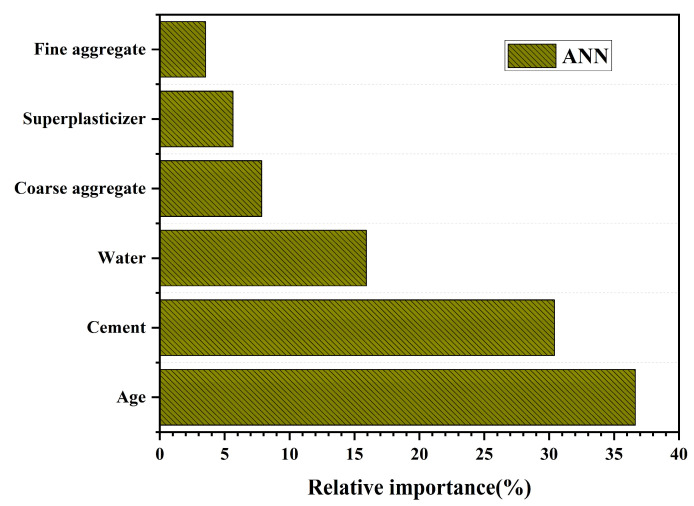
Feature Importance Analysis Based on ANN Model.

Figure 14 presents the results of feature importance analysis derived from the ANN model. The choice of ANN for evaluating input variable importance is primarily attributed to its predictive performance and unique advantages observed in this study. During training, ANN relies on adaptive adjustment of weight parameters, which are iteratively optimized via the backpropagation algorithm. This enables the network to not only identify the contribution of individual variables but also capture interaction effects among them [65,66].

In this study, ANN demonstrated strong predictive accuracy. Although GPR shows slightly better robustness on the testing set, ANN performs well on the training set. It achieves an R^2^ of about 0.96, an RMSE of around 3.9, and an MAE of about 2.5. These results indicate that ANN can effectively fit the characteristic patterns of the data. With relatively large sample sizes, the advantages of ANN become more pronounced, as it can rapidly capture deeper patterns through iterative optimization. Consequently, applying ANN to feature importance analysis provides detailed and reliable insights into the relative contributions of different variables [66,67]. As shown in Figure 14, Age and Cement emerge as the two most influential variables, contributing 36% and 30%, respectively, together accounting for more than 65% of the total importance. This highlights that compressive strength is primarily governed by curing time and the amount of cementitious material. Water contributes about 16%, underscoring the role of the water-to-binder ratio in regulating structural compactness and porosity. The remaining variables (Coarse aggregate, Superplasticizer, and Fine aggregate) together contribute less than 20%, indicating that their impact on compressive strength prediction is relatively minor.

### 3.5. Sustainability Assessment and Environmental Impact Analysis of the Concrete Mixture Composition

After predicting the compressive strength of ultra-high-performance concrete with machine learning models, it is equally important to analyze the sustainability dimension. ultra-high-performance concrete must not only meet mechanical performance but also confront the significant environmental burdens associated with its production and use, including embodied CO_2_ emissions, energy consumption, and resource depletion [66,68]. By assessing the environmental impacts of different mix proportions, this section aims to elucidate how mix design simultaneously affects both strength development and sustainability performance. The main contribution to compressive strength arises from the formation of calcium silicate hydrate (C–S–H) during the hydration of tricalcium and dicalcium silicates, which densifies the microstructure and enhances mechanical performance. The clinker content primarily determines the baseline of embodied CO_2_ emissions, since the calcination of limestone and the high-temperature burning process during clinker production are the dominant sources of carbon in Portland cement. Such an analysis is instrumental in identifying the trade-offs between structural performance and ecological impact, thereby providing valuable guidance for optimizing sustainable ultra-high-performance concrete mixtures.

(1) Embodied CO_2_ (kg):

This variable represents the carbon dioxide emissions generated during the production of ultra-high-performance concrete materials, expressed in kilograms (kg). It is obtained by calculating the carbon emissions of each raw material during the production process, using industry-average emission factors for estimation.

(2) Energy Consumption (MJ):

Energy consumption refers to the total energy required for ultra-high-performance concrete production, expressed in megajoules (MJ). Similarly to embodied CO_2_, it is estimated by calculating the energy consumed during the production of each constituent material. The energy consumption of each raw material is determined based on industry-standard consumption factors.

(3) Resource Consumption (kg):

Resource consumption indicates the total mass of all raw materials used in the production of ultra-high-performance concrete, expressed in kilograms (kg). It is calculated as the sum of all components, including cement, water, aggregates, and superplasticizers. This variable reflects the total amount of resources required to produce one cubic meter of ultra-high-performance concrete.

(4) Environmental Impact Index (/):

To quantify the environmental impact of different ultra-high-performance concrete mixtures, this study introduces the Environmental Impact Index (EII), which integrates three major environmental indicators: embodied CO_2_, energy consumption, and resource consumption. The EII provides a comprehensive and quantitative assessment of the environmental sustainability of ultra-high-performance concrete production. It is calculated by standardizing the three environmental indicators and combining them through a weighted summation to obtain an overall score. The specific formula is defined as follows:

(5) EII=Embodied CO2Max(Embodied CO2)+Enery concumptionMax(Enery concumption)+Resource concumptionMax(Resource concumption)

To eliminate the influence of differences in magnitude among these indicators, all environmental impact metrics were normalized to the range [0, 1]. The normalization was performed by dividing the value of each indicator by the maximum observed value of that indicator. This approach enables a fair comparison of all environmental impact factors on a unified scale. The final Environmental Impact Index (*EII*) was obtained by summing the normalized values of the three indicators. Higher *EII* values indicate a greater environmental burden, implying that the corresponding ultra-high-performance concrete mix design exerts a larger negative impact on the environment. By employing *EII*, it becomes possible to quantitatively assess the environmental impact of different ultra-high-performance concrete mixtures, thereby providing a rational basis for optimizing mix design and reducing environmental burdens. In practice, *EII* not only facilitates the evaluation and comparison of sustainability across different ultra-high-performance concrete mixes but also serves as a valuable tool to guide the development of environmentally friendly construction materials and the optimization of resource use during ultra-high-performance concrete production [69,70].

**Figure 15:**
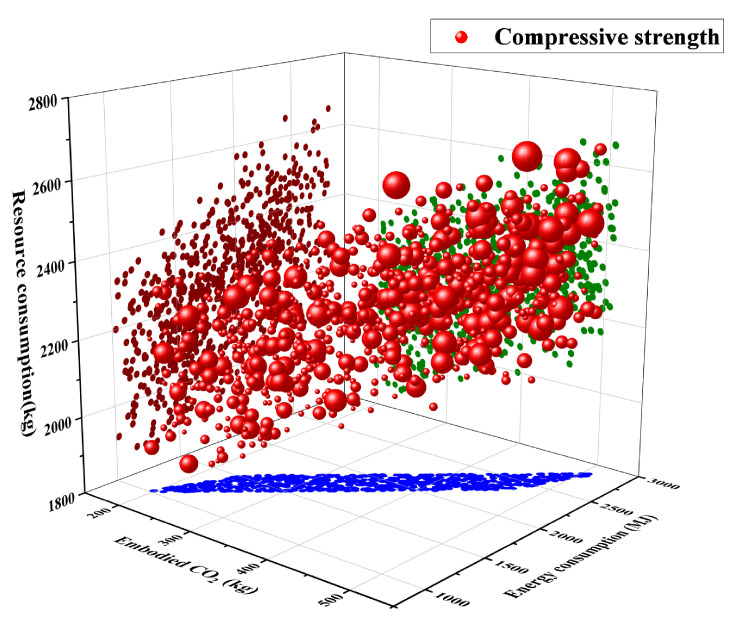
Three-dimensional bubble plot of compressive strength versus CO_2_ emissions, energy consumption, and resource consumption (bubble size indicates strength).

Figure 15 illustrates the relationship between compressive strength and three environmental indicators (embodied CO_2_ emissions, energy consumption, and resource consumption). The x-, y-, and z-axes represent embodied CO_2_ emissions, energy consumption, and resource consumption, respectively, while the size and color of the bubbles indicate the corresponding compressive strength and environmental performance levels. In the plot, red bubbles represent compressive strength, with bubble size proportional to the strength values. Dark red, green, and blue points map the distribution of materials under different environmental levels. This three-dimensional scatter plot provides an intuitive visualization of the interaction between mechanical performance and environmental indicators. Compressive strength increases progressively with higher CO_2_ emissions, energy use, and resource inputs, showing a clear positive correlation between mechanical performance and environmental impact.

From the perspective of resource consumption, red bubbles are mainly concentrated in the range of 2000–2600 kg. Both the number and size of bubbles increase markedly, indicating that compressive strength tends to rise with higher resource inputs. For instance, when resource consumption approaches 2600 kg, a large number of large red bubbles emerge, corresponding to higher compressive strengths. Conversely, below 2000 kg, the bubbles are sparse and relatively small, suggesting that low resource input cannot sustain high performance. The higher cementitious material content increases hydration products. This improves structural compactness and strength but also increases material consumption [71]. The enhanced strength is attributed to the higher cementitious content, which promotes hydration reactions and improves microstructural compactness, although it inevitably increases both material and energy consumption.

A similar trend is observed for embodied CO_2_ emissions. CO_2_ emissions increase from 2000 to 5000 kg, and bubbles grow larger. This means that higher strength causes more carbon emissions. Likewise, the relationship between energy consumption and compressive strength is evident. Larger red bubbles are predominantly located in the high-energy region (1400–2000 MJ), whereas in the low-energy region (around 1000 MJ), bubble size shows little growth. The color distribution illustrates that red points correspond to mixtures with higher environmental impacts and strengths, green points represent moderate conditions, and blue points are associated with lower impacts and lower strength levels.

Blue points cluster mainly in regions of low resource use, low energy consumption, and low CO_2_ emissions, and are generally associated with lower compressive strengths. Green points are scattered across medium levels, where some samples achieve moderate strength under moderate environmental impacts. This indicates that although the general trend is an increase in performance accompanied by higher environmental burdens. It is possible to design mixtures that balance mechanical performance with environmental considerations.

Figure 15 highlights the inherent balance between performance and sustainability. On one hand, achieving high-strength ultra-high-performance concrete almost inevitably requires greater resource consumption and environmental cost [72,73]. Increasing the clinker ratio accelerates hydration and improves early-age strength, but the marginal strength gain diminishes as the associated energy consumption and carbon emissions increase disproportionately with higher clinker usage. On the other hand, prioritizing low-carbon and low-energy designs often compromises mechanical performance. However, high-strength concrete can significantly improve structural durability and service life, thereby reducing maintenance and reconstruction demands over the lifecycle, which in turn mitigates additional energy use and carbon emissions. The results highlight the inherent balance between mechanical performance and sustainability, suggesting that optimized UHPC mixtures can achieve high strength while mitigating environmental burdens through efficient material design. Future material development and engineering applications should increasingly focus on balancing performance and sustainability to achieve the dual goals of structural reliability and environmental friendliness [74,75,76].

**Figure 16:**
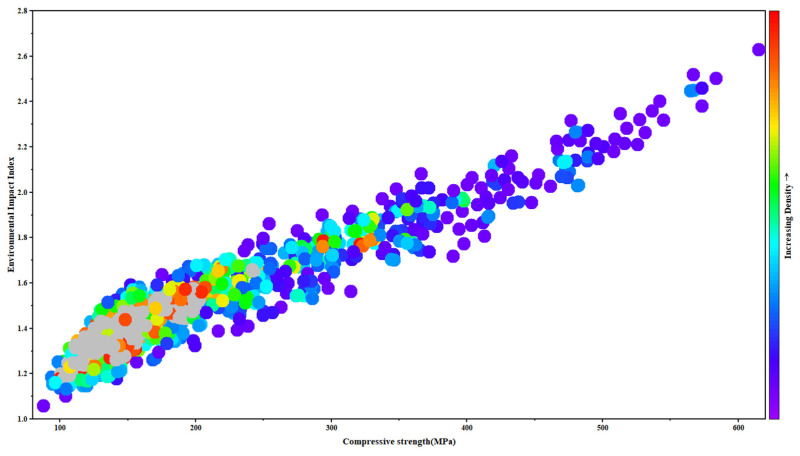
Scatter density distribution of ultra-high-performance concrete compressive strength in relation to the environmental impact index.

Figure 16 depicts the correlation between compressive strength and the Environmental Impact Index (EII). The two exhibit a strong positive correlation. As compressive strength increases from 100 MPa to 600 MPa, the EII rises from approximately 1.1 to above 2.6, displaying a nearly linear growth trend. This finding indicates that improvements in material performance are consistently accompanied by increased environmental burdens, highlighting the inherent trade-off between strength and sustainability [77].

From the perspective of data density distribution, distinct environmental costs are associated with different strength intervals. In the 150–250 MPa range, data points are most densely clustered, predominantly colored in red and orange, with EII values concentrated between 1.3 and 1.7. This interval represents a relatively balanced region, where adequate structural performance can be achieved while keeping environmental impacts at a moderate level. As compressive strength increases to the 300–450 MPa range, data density decreases and colors shift toward blue-green, with EII values rising significantly to the 1.7–2.2 range. This suggests that further strength gains require additional consumption of high-performance cementitious materials, leading to pronounced increases in CO_2_ emissions and energy consumption [78,79]. When compressive strength exceeds 500 MPa, the number of data points drops sharply, colors turn bluish-purple, and the EII surges above 2.5. This indicates that although high-strength ultra-high-performance concrete offers mechanical advantages, its production entails extremely high energy use and carbon emissions, making it suitable only for specialized engineering applications or extreme service conditions [80,81].

This trend is consistent with the micromechanical mechanisms of ultra-high-performance concrete. Achieving higher strength necessitates increased dosages of cement and reactive auxiliary cementitious materials together with optimized water-to-binder ratios, processes that inevitably intensify resource use, energy demand, and CO_2_ emissions [81]. The optimal balance between strength and sustainability is achieved through maximizing binder efficiency rather than simply increasing the total binder content, as improved hydration kinetics, particle packing, and curing regimes can yield comparable strength at lower environmental cost. Therefore, the strength range of 150–250 MPa may be considered an optimal balance point between mechanical performance and sustainability. Ultra-high-performance concrete can meet the structural requirements of most engineering applications while maintaining a reasonable environmental footprint.

## 4. Conclusions

Based on a dataset of 800 samples, this study assessed four machine learning models (RF, GPR, GB, and ANN) for predicting compressive strength and examined the relationship between strength and environmental impact. The major findings of this study can be summarized as follows:

1. All machine learning models demonstrated strong predictive capability. The predicted values agree well with the experimental results. Most data points fall within a ±10% error range, confirming the effectiveness of data-driven approaches for compressive strength prediction.

2. GPR and ANN achieved the best performance but through different mechanisms. ANN reached high accuracy through nonlinear mapping. GPR showed stronger robustness and uncertainty quantification, making it ideal for reliable prediction tasks.

3. The GB model offered a good balance between accuracy and generalization, with R^2^ values consistently around 0.90 and moderate error levels across both training and testing sets. GB showed strong adaptability and is promising for applications under varying data scales and conditions.

4. The RF model exhibited limitations in high-strength ranges. Although it captured the overall trend, prediction errors increased under extreme conditions. The R^2^ value on the testing set dropped to 0.82. Therefore, RF is more suitable as a complementary reference model than as the primary predictor.

5. Key variable influences were evident. Feature importance analysis revealed that curing age and cement content were the dominant factors, jointly contributing over 65% to compressive strength prediction. Water-to-binder ratio, reflected through the water variable, played an indirect role, while the direct contributions of other variables were relatively limited.

6. There is a balance between performance and sustainability. Results showed that strength improvement is generally accompanied by increases in CO_2_ emissions, energy use, and resource consumption. The 150–250 MPa strength range was identified as the optimal balance zone, achieving adequate structural performance while maintaining reasonable environmental costs.

## Figures and Tables

**Table 1:** Variable names, units, and descriptions.

Variables	Unit	Description
Cement	kg/m^3^	The amount of cement in the ultra-high-performance concrete
Superplasticizer	kg/m^3^	The addition of superplasticizer improves workability without the need for additional water.
Water	kg/m^3^	The water content of ultra-high-performance concrete
Coarse aggregate	kg/m^3^	The quantity of coarse aggregates such as gravel or crushed stones
Age	Day	The curing time of the samples
Fine aggregate	kg/m^3^	The quantity of fine aggregates such as sand
Compressive strength	MPa	The strength of ultra-high-performance concrete under compression

**Table 2:** Statistics of the dataset.

Parameters	Mean	Max	Std. Dev	Min	Median	Var	Skewness	Kurtosis	SE	Range
Cement	348.56	499.92	87.96	201.39	350.81	7737.74	0.02	−1.23	3.11	298.53
Superplasticizer	14.99	29.93	8.71	0.00	14.86	75.87	−0.01	−1.18	0.31	29.93
Water	185.44	219.96	20.43	150.35	186.36	417.29	−0.06	−1.22	0.72	69.61
Coarse aggregate	948.28	1099.87	85.56	800.20	947.11	7320.02	0.01	−1.21	3.02	299.67
Age	180.46	362.00	104.27	1.00	182.00	10,871.51	0.02	−1.19	3.69	361.00
Fine aggregate	746.18	899.32	85.97	600.01	745.50	7391.01	0.06	−1.18	3.04	299.32
Compressive strength	225.23	615.21	108.62	87.83	189.19	11,798.87	1.17	0.69	3.84	527.38

## Data Availability

The data used in this study are not publicly available due to institutional restrictions but can be obtained from the corresponding authors upon reasonable request.
